# Resident and phytometer plants host comparable rhizosphere fungal communities in managed grassland ecosystems

**DOI:** 10.1038/s41598-020-57760-x

**Published:** 2020-01-22

**Authors:** Ricardo Schöps, Kezia Goldmann, Lotte Korell, Helge Bruelheide, Tesfaye Wubet, François Buscot

**Affiliations:** 1 UFZ – Helmholtz Centre for Environmental Research, Department of Soil Ecology, Theodor-Lieser-Straße 4, 06120 Halle/Saale, Germany; 2 University of Leipzig, Department of Biology II, Johannisallee 21, 04103 Leipzig, Germany; 3 Martin Luther University Halle-Wittenberg, Institute of Biology/Geobotany and Botanical Garden, Am Kirchtor 1, 06108 Halle/Saale, Germany; 4 UFZ – Helmholtz Centre for Environmental Research, Department of Community Ecology, Theodor-Lieser-Straße 4, 06120 Halle/Saale, Germany; 5 German Centre for Integrative Biodiversity Research (iDiv) Halle-Jena-Leipzig, Deutscher Platz 5e, 04103 Leipzig, Germany

**Keywords:** Microbial ecology, Grassland ecology

## Abstract

Plants are known to modulate their own rhizosphere mycobiome. However, field studies that use resident plants to relate the microbiome assemblage to environmental factors such as land-use suffer from the problem that confounding factors such as plant age and performance may override the targeted effects. In contrast, the use of even-aged phytometer plants pre-cultivated under uniform conditions helps to reduce such random variation. We investigated the rhizosphere mycobiomes of phytometer and resident plants of two common grassland species, *Dactylis glomerata* L. s. str. and *Plantago lanceolata* L. along a land-use intensity gradient using ITS rRNA Illumina amplicon sequencing. Remarkably, we did not detect effects of the plant types (resident vs. phytometer plant, even though some fungal taxa exhibited plant species specificity), indicating that phytometer plants hosted a comparable rhizosphere mycobiome as resident plants. Our data indicate that the plant species harbor distinct fungal communities, with fungal richness in the rhizosphere of *P. lanceolata* being substantially higher than that of *D. glomerata*. Land-use intensity had a clear impact on the mycobiome of both plant species, with specific fungal genera showing differential tolerance to high intensities. Overall, the phytometer approach has a high potential to reveal environmental impacts on rhizosphere communities.

## Introduction

The plant rhizosphere – the soil attached to or located in close proximity to plant roots – is known as a hotspot of microbial activity and diversity^1^. One highly diverse and active microbial group in the rhizosphere are fungi including species that have positive as well as adverse effects on the plant growth, nutrition and health^2^. Plants can actively shape and select their rhizosphere mycobiome by secretion of photosynthates and release of root exudates^3^. The amount and type of root exudates as well as root morphology are plant species-specific^4,5^ and contribute to plant species identity effects on fungal communities^6^. The surrounding bulk soil, i.e. soil not in close contact to plant roots serves as source or propagule bank of fungi that can potentially be selected by growing roots^7^. Studies carried out in grasslands under realistic field conditions have mostly examined the rhizosphere soil communities of resident plants^6,8^. With resident plants, we refer to those plants that have established themselves in the field from seeds of the regional species pool. Such resident plants have accessed the bulk microbial propagule bank since their germination, and thus may have established their particular rhizosphere mycobiome. However, the rhizosphere soil of resident plants may be prone to unwanted variations superimposing the plant identity effects because the plant age and the conditions may strongly vary among individuals. One way to avoid such variation is to use standardized phytometer plants introduced in the field. Phytometers are transplanted individuals of plant species that were raised under uniform conditions from homogeneous seed populations and are of the same age^9–11^. Thus, phytometers are a suitable method to eliminate unwanted confounding variation in field studies. Furthermore, under realistic field conditions, also widely distributed resident plant species with a high abundance in the study region may not necessarily be found in all target experimental plots^12^ aggravating the choice of repetitions and leading to an un-balanced study design. However, transplanted phytometer plants must first establish themselves and trap their rhizosphere mycobiome from the resident soil communities, for which a certain time is required. It has been shown previously that six months in the field may be sufficient to develop a rhizosphere mycobiome different to the bulk soil^12^. Recently, Chung, *et al*.^13^ showed that roots of the grasses *Bouteloua gracilis* and *B. eriopoda* which had been raised in the greenhouse and then transplanted to the field had substantially different fungal communities compared to plants naturally occurring in the field. Additionally, in their study transplanted plants had lower among sample variability in community composition and hosted a higher fungal diversity. Thus, it is possible that the fungal communities in the rhizosphere of phytometer plants could be distinct from those of the resident plants and display lower variation, e.g. because of difference in establishment time. However, there are no studies yet on rhizosphere communities that have addressed this question.

Studies on the effects of environmental conditions on rhizosphere communities commonly focus on soil conditions^14^, disturbances such as climate extremes^15^, and on primary or secondary vegetation succession^16,17^. Yet, it is largely unknown to which degree rhizosphere communities of typical grassland species are affected by a gradient of land-use intensity (determined by the degree of mowing, fertilization, and grazing) within the same ecosystem^12^. Land-use intensification is known to homogenize belowground communities likely by decreasing microhabitat quality and complexity^18^. Moreover, it was also shown that the abundance of dominant fungal genera was highly dependent on land-use intensity with only a few genera showing land-use optima at high intensities, while most genera were present at intermediate and low land-use intensities^12^. Therefore, intensive land-use may lead to population decline of most fungal genera. By defining a ‘land-use niche′ and a corresponding ‘niche breadth′ for fungal genera, we can distinguish between losers and winners of intensive land-use as well as determine specialists adapted to intermediate land-use^19^. In addition, it is of interest, whether fungal genera react differently to land-use intensity depending on residence time, i.e. comparing resident vs. phytometer plants.

Here we provide the first comparison of rhizosphere fungal communities between phytometer plants and established resident plants of grassland plant species. We assessed the effects of plant type (phytometer vs. resident) across two common plant species (*Dactylis glomerata* L. s. str. (grass) and *Plantago lanceolata* L. (forb)) along a land-use gradient in 13 temperate grassland plots by employing paired-end Illumina sequencing of the fungal internal transcribed spacer (ITS2) region. Using a gradient design we avoid pseudo-replication (i.e. by having more than one sample in each experimental plot) and these multisite observations enable us to draw more general conclusions about the role of plant type and plant species across different background environmental conditions^20^. We hypothesized that (i) the rhizosphere mycobiomes are distinct between the plants species because of differences in their root exudation and other plant traits. We further predicted that (ii) plant type influences the fungal community as the resident plants have more time to establish connections to the surrounding bulk soil which lead to an enriched community compared to phytometers or to a reduced diversity by selecting only specific fungi. Therefore, the fungal community composition also differs between the plant types and resident plants have greater variation among individuals in their fungal composition. Finally, (iii) we expected for both, resident and phytometer plants, a higher number of dominant fungal genera to be negatively influenced by land-use intensification (high land-use intensity).

## Results

### Bioinformatics and taxonomy

The fungal community profiling yielded a total of 2,440,337 high-quality sequences (range 27,468-66,115; median 46,396) from 52 samples. We identified 22,641 fungal OTUs across all samples, ranging between 532 and 3,722 OTUs per sample (median 1,544). We were able to assign 97, 91, 90, 54, 72, and 58% of the total fungal OTUs at phylum, class, order, family, genus, and species levels, respectively. Taxonomic assignments resulted in a distribution of OTUs across nine phyla with a marked dominance of Ascomycota with 84% of all OTUs, followed by 10% Basidiomycota, 2% Glomeromycota, and others (divided in Mortierellomycota 0.5%, Chytridiomycota 0.4%, Rozellomycota 0.2%, Mucoromycota 0.09%, Kickxellomycota 0.04%, and Basidiobolomycota 0.004%) as well as 3% of OTUs that could not be classified. At the order level, OTUs were classified into 85 orders, of which Hypocreales (23%), Capnodiales (17%), Pleosporales (14%), and Xylariales (11%) dominated in terms of OTU richness. Mycosphaerellaceae (15%) and Nectriaceae (14%) were the most dominant families. At the genus level, rhizosphere fungi were dominated by *Mycosphaerella* (15%), *Gibberella* (12%), and *Monographella* (9%).

### Fungal α-diversity

**Figure 1:**
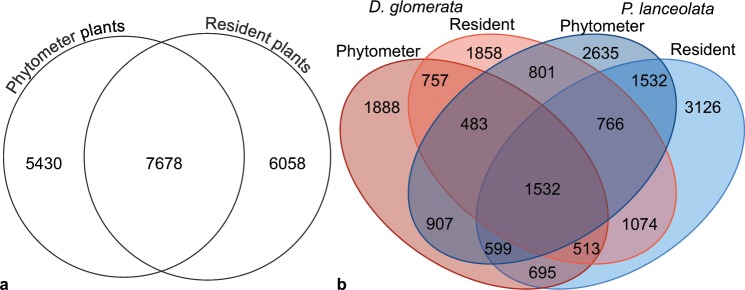
Numbers of fungal OTUs shared between (**a**) the plant type (phytometer vs. resident plants) and (**b**) among plant species (*Dactylis glomerata* and *Plantago lanceolata*) and plant type.

**Figure 2:**
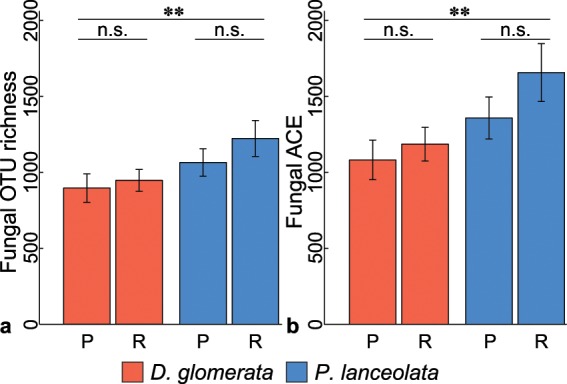
Differences in alpha-diversity measured using (**a**) observed OTU richness and (**b**) estimated abundance-based coverage estimator (ACE) between plant species (*Dactylis glomerata* (red) and *Plantago lanceolata* (blue)) and plant type (P, phytometer and R, resident plants). Mean values for each plant type and plant species are plotted with ± standard error. Asterisks indicate strong differences between plant species (observed and estimated OTU richness *P* < 0.006 and *P* < 0.003, respectively; Supplementary Table S1). n.s., no strong effect of plant type (*P* > 0.05).

For α-diversity analyses, we normalized the fungal communities to 27,468 sequences per sample. From the 22,641 OTUs, we retained 19,116 fungal OTUs, ranging between 434 and 1,992 OTUs per sample (median 991). Overall, 7,678 OTUs of the 19,116 fungal OTUs were shared (40%) between the plant types (Fig. 1a), while 6,058 OTUs (32%) were unique in the resident and 5,430 OTUs (28%) in the phytometer plants, respectively. We detected more plant species-specific OTUs in the rhizosphere soil of *P. lanceolata* (7,293 OTUs) than in the one of *D. glomerata* (4,503 OTUs, Fig. 1b). Of these plant species-specific OTUs, a slightly higher proportion was shared between the resident and phytometer plants of *P. lanceolata* (1,532/7,293 OTUs, 21%) as compared to the plant types of *D. glomerata* (757/4,503 OTUs, 17%). Interestingly, a low proportion of fungal OTUs was shared between the plant species and plant types (1,532 OTUs, 8%; Fig. 1b). At plant species level (Supplementary Fig. S1), fungal OTUs that were shared between the plant types represented a lower proportion (28% and 30% in *D. glomerata* and *P. lanceolata*, respectively) compared to the unique OTUs in the phytometer plants (34% in *D. glomerata* and 33% *P. lanceolata*) and in the resident plants (38% in *D. glomerata* and 37% *P. lanceolata*). By subtracting the plant species-specific OTUs from the phytometer plants, resident plants, and shared sets of *D. glomerata* or *P. lanceolata* (Supplementary Fig. S1), the high proportion of shared OTUs between phytometer and resident plants was retained for both plant species. In total, the median fungal OTU richness and abundance-based coverage estimator (ACE) was 991 (ranging from 434 to 1,992 OTUs) and 1,239 (ranging from 475 to 3,054 OTUs), respectively. We found that fungal observed and estimated OTU richness in the rhizosphere of *P. lanceolata* were clearly higher than in *D. glomerata* (*P* < 0.01; Fig. 2a,b; Supplementary Table S1). There was no influence of the plant type on fungal α-diversity. We also found no interaction between plant species and plant type (Supplementary Table S1).

### Fungal community composition (β-diversity)

**Figure 3:**
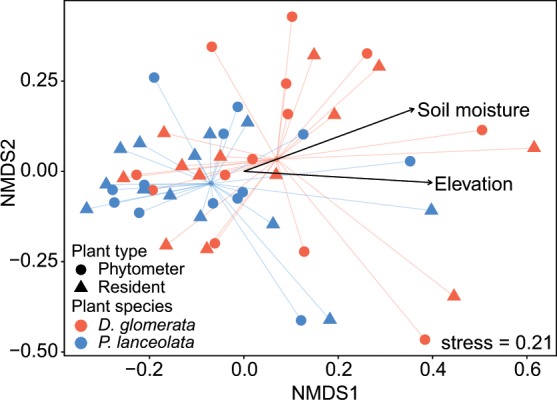
Non-metric multidimensional scaling (NMDS) ordination of fungal community composition among different plant species and plant types. The NMDS was based on Bray-Curtis dissimilarity and k = 2 dimensions. Only factors that were strongly correlated (*P* < 0.05) with community composition are shown.

Differences in fungal community composition were evaluated using permutational multivariate analysis of variance (PERMANOVA), which indicated that each plant species harbored a different fungal rhizosphere community (pseudo *F* = 1.94; *P* = 0.002; Table 1). In addition, LUI strongly affected total fungal community composition (pseudo *F* = 2.21; *P* = 0.001) and the fungal community of the resident (pseudo *F* = 1.40; *P* = 0.049) and of the phytometer plants separately (pseudo *F* = 1.48; *P* = 0.036). *D. glomerata* was strongly (pseudo *F* = 1.61; *P* = 0.010) and *P. lanceolata* was slightly, but still significantly (pseudo *F* = 1.37; *P* = 0.026) influenced by LUI. Resident and phytometer plants harbored comparable fungal communities (pseudo *F* = 0.64; *P* > 0.1; Table 1). There was also no plant type effect on fungal community composition when performing separate analysis for *P. lanceolata* (pseudo *F* = 0.71; *P* > 0.1) and *D. glomerata* (pseudo *F* = 0.62; *P* > 0.1; Table 1). Among the topographic features, geographic location, and climate variables, we found that total fungal communities in the rhizosphere were primarily driven by soil moisture (*R²* = 0.12; *P* = 0.043; Fig. 3) and elevation (*R²* = 0.15; *P* = 0.026). Test of multivariate homogeneity of group dispersions showed that variances did not differ between phytometer and resident plants (pseudo *F* = 0.027; *P* = 0.872).

**Table 1:** Effect of land-use intensity (LUI), plant species, and plant type on fungal community compositions assessed with permutational multivariate analysis of variance (PERMANOVA).

Fungi	Total				Phytometer plants				Resident plants				*D. glomerata*				*P. lanceolata*			
	df	*F*	*P*	*R*^2^	df	*F*	*P*	*R*^2^	df	*F*	*P*	*R*^2^	df	*F*	*P*	*R*^2^	df	*F*	*P*	*R*^2^
Land-use intensity (LUI)	1	2.21	**0.001**	0.04	1	1.48	**0.036**	0.03	1	1.40	**0.049**	0.05	1	1.61	**0.010**	0.06	1	1.37	**0.026**	0.06
Plant species (PS)	1	1.94	**0.002**	0.04	1	1.19	0.172	0.15	1	1.47	**0.032**	0.06								
Plant type (PT)	1	0.64	0.993	0.01									1	0.62	0.996	0.03	1	0.71	0.955	0.03
LUI × PS	1	1.00	0.961	0.02	1	0.71	0.930	0.03	1	1.03	0.471	0.04								
LUI × PT	1	0.65	0.996	0.01									1	0.73	0.939	0.03	1	0.64	0.953	0.03
PS × PT	1	0.69	0.996	0.01																
LUI × PS × PT	1	0.71	0.996	0.01																
Residuals	44			0.85	22			0.87	22			0.85	22			0.88	22			0.88

Plant species (*Dactylis glomerata* vs. *Plantago lanceolata*); plant type (phytometer vs. resident plants); df, degrees of freedom; *F* and *P*, pseudo *F*-statistics and *P*-values; Strong differences are indicated in bold (*P* < 0.05); *R²*, partial coefficient of determination.

### Losers, mid-specialists, and winners of high land-use intensity

**Figure 4:**
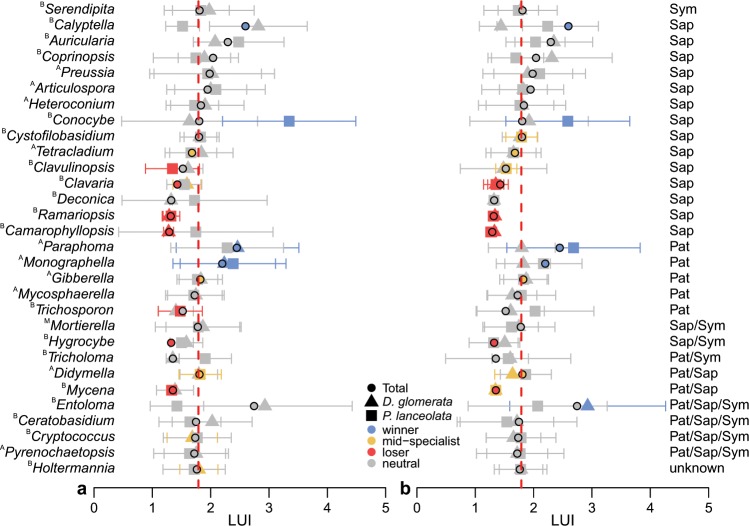
The abundance-weighted means (*μ*_*j,obs*_) and standard deviations (*σ*_*j,obs*_) of land-use intensity (LUI; consisting of the land-use elements grazing, mowing, and fertilization) are shown for each of the top 30 fungal genera (Supplementary Table S3) for (**a**) phytometer and (**b**) resident plants. Dotted lines illustrate the mean land-use intensity for the interval 2006-2014 across all experimental plots and it is representative of the expected weighted mean value from the null model based on 10,000 randomizations. Fungal trophic modes: Sym – symbiotroph, Sap – saprotroph, Pat – pathotroph, Sap/Sym – saprotroph/symbiotroph, Pat/Sap – pathotroph/saprotroph, Pat/Sap/Sym - pathotroph/saprotroph/symbiotroph. ^A^, ascomycetous genus; ^B^, basidiomycetous genus; ^M^, Mortierellomycota.

From the Similarity percentages (SIMPER) analysis using the entire dataset, the top 30 fungal genera that contributed the most to the observed overall dissimilarity between the plant types (phytometer vs. resident plants) were taken for the analysis of their ‘land-use niche′ (Supplementary Table S2). Of the top 30 fungal genera, a majority of 14 genera were functionally assigned to the trophic mode saprotroph (Fig. 4, Supplementary Table S3) using FUNGuild^21^. In addition, five and one of the genera were clearly categorized as pathotroph and symbiotroph, respectively. The remaining genera were not clearly assigned to a specific trophic mode (two genera saprotroph/symbiotroph, two saprotroph/pathotroph, one pathotroph/symbiotroph, and four pathotroph/saprotroph/symbiotroph) or the trophic mode was unknown (one genus). In general, most genera reacted neutrally to intensive land-use and the full spectrum of land-use niches occurred (‘winners′, ‘mid-specialists’, and ‘loser’) within the top 30 fungal genera (Fig. 4; Supplementary Table S3). Five genera were losers of high LUI, whereas three genera were winners and three genera were mid-specialists. Differences became visible between the individual treatments of plant types and species, e.g. *Mycena* was a mid-specialist in the rhizosphere soil of the resident plants, while it was a loser of intensive land-use in total and separately in phytometer plants. Similarly, the pathotrophic genus *Monographella* showed differences and was unaffected by intensive land-use in the resident plants, whereas apparently it had an advantage among the phytometer plants. In contrast, the saprotrophs *Ramariopsis* and *Camarophyllopsis* were consistently losers of intensive land-use.

## Discussion

Our results demonstrate a different rhizosphere fungal OTU richness between the two plant species. The rhizosphere fungal communities of *P. lanceolata* are more diverse than that of *D. glomerata*. This may reflect a high degree of plant species-specificity^2^. Apart from differences in the OTU richness, we found that the two plant species selected distinct fungal communities in their rhizospheres which provides strong support for our first hypothesis. This is hardly surprising given the number of studies showing that plant species identity strongly shapes the compositions of fungi in the rhizosphere^6,22–24^. Plant species-specific differences in root traits and root exudation as well as effects on soil physicochemical properties, e.g. on nutrient availability^25^ are frequently responsible for this differential diversity and assembly of the rhizosphere mycobiomes^1^. Apart from the core mycobiome that is broadly distributed and can be found in the rhizosphere of numerous plant species, increasing evidence suggests that there are specific root exudate consumers which are unique to a plant species consuming their secreted simple carbon substrates^26^. Together with our results, this indicates that plants play a crucial role in the selection of their rhizosphere consortium. However, in contrast to this general assumption, other studies reported only minor effects of plant species or genotypes on the rhizosphere mycobiomes^27–29^. At least one possible reason for strong plant species and rhizosphere-bulk soil effects seems to be the duration of the experiment, i.e. the time the plant can grow in the soil and establish a specific rhizosphere mycobiome. Indeed, in our previous work, we reported hardly any differences between the fungal rhizosphere communities of four phytometer plant species (including the plant species *D. glomerata* and *P. lanceolata*) after six months growing in the field^12^. This study further assumed that especially with this relatively short duration, the environmental and land-use conditions superimpose any existing plant species identity effect. Likewise, we revealed no plant species effect for the phytometer plants after one year of growth in the field in the present study, and we found only a plant species identity effect for the resident plants. It is tempting to speculate, whether this indicates another time-related dependency to establish specific fungal communities in perennial plant species or rather a dependency on environmental conditions. To support such findings and to draw more general conclusions further studies are needed across a broad range of host plant species, especially under field conditions.

We expected differences between the plant types as the resident plants had the possibility to build up their rhizosphere mycobiome over a longer period of time through constant contact with the initial propagule reservoir of the fungal community^30^. In turn, the shorter exposure time of the phytometer plants in soils of the experimental plots should have provided fewer opportunities to select a plant species-specific rhizosphere mycobiome. Remarkably and in contrast to our second hypothesis, we did not detect any consistent difference between the rhizosphere fungal communities of the resident and phytometer plants. Although there were unique fungal OTUs in the resident (32%) and phytometer (28%) plants, the majority of the detected fungi were shared (40%) and thus the observed/estimated fungal OTU richness was similar between the plant types. Even the variability in community composition between the plant types was extremely low. Our result on fungal communities in the rhizosphere are also in accordance with similar active bacterial rhizosphere communities between resident and phytometer plants^31^. Moreover, a study on the perennial forb *Boechera stricta* (Brassicaceae) in its native range in central Idaho (USA) indicates that root bacterial composition of greenhouse plants transplanted to grassland field sites became more similar to resident control plants (wild growing with unknown age) over time^32^. Wagner, *et al*.^32^ presumed that the potting-soil bacteria present in the roots were replaced by natural ones in the field and thus an equilibrium was reached with the surrounding soils within two years after transplantation. However, as aforementioned, Chung, *et al*.^13^ found separate root mycobiomes between transplants raised in the greenhouse and field residents. This might be dependent on the large age differences as transplants raised in the greenhouse were only for 25 months in the field, while field resident were at least 20 years old^13^. Therefore, we propose that our results should be further verified, e.g. in other plant compartments and temporal discrepancies should always be considered. In conclusion, we found no clear difference in fungal rhizosphere communities between plant types, thus we can clearly recommend the usage of phytometer plants when studying mycobiomes due to their uniform breeding and age^10,33^. Although it is possible to determine the age of resident plants by growth ring analysis after destructive sampling^34^, in grasses as well as specific forb families this method is problematic because of an absence of secondary growth^35^. In addition, in the field it can be difficult to find a batch of plants in a comparable age^36^ based on plant size as proxy for plant age. Size-related differences of resident plants, e.g. due to small-scale differences in soil fertility, may thus potentially obscure treatment effects. It is necessary to include naturally occurring individual variation in age and size if studying effects on, e.g. genetic or demographic differences between plant populations^37^. However, our findings suggest that phytometer plants growing for one year in the field have established a comparable rhizosphere mycobiome as resident plants. The results of phytometer approaches can be considered as realistic and their rhizosphere mycobiomes are congruent to those longer time present under field conditions.

In our study, we detected varying fungal community compositions in the rhizosphere with changing LUI. This is consistent to our previous study on the bulk and rhizosphere soil community in the Hainich-Dün region^12^. Interestingly, the fungal community in *D. glomerata* was slightly more affected by LUI than the fungal community in the rhizosphere of *P. lanceolata*. For arbuscular mycorrhizal fungi, it was shown that LUI and host plant identity can interactively shape communities^38^. Hence, *D. glomerata* might be more sensitive to disturbances such as mowing or grazing compared to *P. lanceolata*. Generally, land-use intensification can have strong effects on soil pH, quality and quantity of nutrients, and soil structure^39,40^, and such effects can drive fungal communities associated with plants^12,41,42^. The rhizosphere mycobiome may, however, also respond indirectly to LUI via disturbance of their plant host by mowing or grazing activity as shown for culturable root-associated fungi^43^. To analyze the effects of LUI in detail, we characterized the ‘land-use niche’ and the ‘niche breadth’^19,44^ of the top 30 fungal genera. In general, the patterns of the top 30 fungal genera in their response to the LUI were considerably consistent between phytometer and resident plants. Additionally, the differentiation between losers and winners of high LUI and mid-specialists also shows some similarities between the plant types, e.g. *Camarophyllopsis*, *Hygrocybe*, and *Ramariopsis*, were found to be losers in the rhizosphere of both plant types at high LUI. These genera are known for their sensitivity to management practice and their preference for unfertilized grasslands^45–47^. Consequently, these genera are important bioindicators in terms of nature conservation^48^ and their occurrence in grasslands point to a generally rich mycobiome^49^. Moreover, *Conocybe* species were winners of land-use intensification mainly in the rhizosphere of *P. lanceolata*. Members of this genus can be found even in strongly fertilized grasslands^45,48^. In contrast, the genus *Mycena* is impaired by intensive land-use in total, but in the rhizosphere of the resident plants it was an intermediate specialist favoring medium land-use. Several *Mycena* species are typical litter saprotrophs^50^, which are possibly well-established in the rhizosphere of resident plants when LUI is intermediate. Furthermore, Monographella including important plant pathogens^51^ was neutral in the rhizosphere of the resident plants and a winner in the phytometer plants. Through the transplantation and still ongoing adaptation processes, the performance of phytometers could be even more affected by the human impact than that of resident plants^33^, which in turn may have positive effects on some of the pathotrophs in the rhizosphere. This is highly speculative and further investigations are needed to disentangle the exact relationships among land-use, plant type, and fungal rhizosphere genera.

In conclusion, although the phytometer plants have been exposed to the field conditions only for one vegetation season, its rhizosphere mycobiome is highly comparable to that of the resident plants. This justifies the further usage of this method for examining the general plant root-associated microbial communities. Our findings support the previous observation that the assemblage of the fungal rhizosphere communities is actively driven by the plant species most probably through the secretion of root exudates. Apart from that, land-use is another major factor that can lead to differences in the abundance of specific fungal genera in rhizosphere soils.

## Methods

### Study area and land-use intensity

This study was conducted in 2015 in the biosphere reserve Schwäbische Alb (ca. 422 km²; 48°43′N9°37′E, 460–860 m above sea level, 700–1000 mm annual precipitation, 6–7 °C mean annual temperature, soil pH 6.23 ± 0.40; mean ± SD) located in a low mountain range in southwest Germany. The sampled sites are part of the German Biodiversity Exploratories project (www.biodiversity-exploratories.de)^52^. We chose 13 experimental grassland plots with different land-use intensities, depending on whether they were either mown, grazed by livestock or a combination of both, and are either unfertilized or fertilized at different intensities^53^. Each experimental plot *i* is specified by a land-use intensity (LUI) index, which combines the three land-use elements (mowing, grazing and fertilization^53^) and is calculated as

1 $$LU{I}_{i}=\sqrt{\frac{{F}_{i}}{{F}_{R}}+\frac{{M}_{i}}{{M}_{R}}+\frac{{G}_{i}}{{G}_{R}}}$$

where intensity of fertilization (*F*_*i*_ in kg N ha^−1^yr^−1^), mowing frequency (*M*_*i*_ in cuts yr^−1^), and grazing intensity (*G*_*i*_ in livestock units days of grazing ha^−1^yr^−1^) are standardized by the mean values of *F*_*R*_, *M*_*R*_, *G*_*R*_ within the Schwäbische Alb region *R*. The square root transformation was applied to produce a more even distribution. We used the mean LUI values for the interval 2006-2014 (ranging from 1.25–3.62, median 1.62; Supplementary Table S4) to both capture the past land-use and the current conditions under which the phytometers were growing in the field. Low and high index values indicate less and more intensive land-use management, respectively. Microclimate stations in each experimental plot measured relative humidity at 200 cm aboveground, soil moisture at 10 cm depth, and air temperature at 10 and 200 cm aboveground. The monthly mean values were extracted of these variables for the 13 experimental plots and the mean values from May 2014 to July 2015 were calculated (see Supplementary Table S4).

### Preparation of the phytometer plants and sample collection of the phytometer and resident plants

The phytometer plants (*Dactylis glomerata* L. s. str. (a perennial grass) and *Plantago lanceolata* L. (a perennial forb)) were grown from seeds collected from grasslands in the Schwäbische Alb region in September (grass) and in July (forb) 2013. We surface-sterilized the seeds in 70% ethanol for 30 s, then in 1% NaClO for 1 min, and finally washed them gently in Milli-Q water three times. The seeds were germinated in 5.5 cm × 5.5 cm pots containing a 1:1 sterilized silt and sand mixture in December 2013 in the greenhouse of the Botanical Garden of the Martin-Luther University in Halle (Saale), Germany. Seedlings were transplanted randomly as phytometers into the 13 experimental plots in early June 2014 (see^10,33^). We used phytometer plant approach^9^ to diminish the random variation by using plants of the same age that have been raised under uniform conditions from homogeneous seed populations^10,11,33^. Each individual of the two plant species planted into each of the 13 experimental plots was harvested after approximately one year (July 2015). In addition, outside of each experimental plot, healthy resident plants of *D. glomerata* and *P. lanceolata*, which if possible were in about the same size as the phytometer plants were harvested for comparison. Here, it should be noted that the size is not a proxy for plant age which was not determined. The phytometer and resident plants were excavated along with monoliths of soil (20 cm × 20 cm × 20 cm) and the rhizosphere soil was taken. In each monolith, the bulk soil was removed by shaking the roots and the soil (rhizosphere) still adhering to the roots was detached by washing thoroughly in 100 ml sterile 0.5% NaClO solution in a 500 ml plastic beaker. We filled both the wash solution with smaller suspended particles and the coarser particles from the bottom into two 50 ml falcon tubes. We then filtered the wash solution through polycarbonate filters (47 mm diameter, 0.2 μm pore size). The remaining coarse soil particles and the polycarbonate filters were combined in 5 ml cryotubes. The cryotubes were immediately flash frozen in liquid nitrogen in the field, and stored on dry ice until their transfer into −80 °C freezers in the laboratory. In total, 52 rhizosphere samples were collected for the analysis (13 experimental plots × 2 plant species (*D. glomerata* and *P. lanceolata*) × 2 plant types (resident and phytometer plants)).

### DNA extraction, library preparation and sequencing

We extracted the total microbial genomic DNA from rhizosphere soils (filters and coarse particles) following the method of Lueders, *et al*.^54^, modified by Wüst, *et al*.^55^. Frozen filter samples were cut into pieces with a sterile pair of scissors and were then transferred together with the coarser soil particles to a 2 ml screw cap tube containing 0.7 g of sterilized zirconium/silica beads (0.1 mm diameter). Subsequently 750 µl sodium phosphate solution (pH 8, 112.9 mM Na_2_HPO_4_ and 7.12 mM NaH_2_PO_4_) and 250 µl TNS-buffer (pH 8, 500 mM Tris-HCl pH 8, 100 mM NaCl, and 10% sodium dodecyl sulfate) were added and the samples were shaken with a TissueLyser II (Qiagen GmbH, Hilden, Germany; at 28 Hz for 4 min). After centrifugation, samples were extracted with phenol-chloroform-isoamyl alcohol (25:24:1) and chloroform-isoamyl alcohol (24:1). PEG (30% polyethylene glycol 6000 in 1.6 M NaCl) was used for DNA precipitation and after centrifugation pellets were washed with cold ethanol (70%) and resuspended in 70 µl buffer EB (Qiagen GmbH, Hilden, Germany). Concentrations of DNA were determined using a NanoDrop 8000 spectrophotometer (Thermo Fisher Scientific, Dreieich, Germany). We generated fungal amplicon libraries by performing semi-nested PCRs to avoid co-amplification of plant ITS sequences. PCRs were performed, starting with amplification of the fungal ITS1 and ITS2 rDNA region using the primer combination ITS1F^56^ and ITS4^57^. Negative controls were performed using the reaction mixture without template. PCRs were carried out using the following parameters: initial denaturation at 95 °C for 5 min, 10 cycles of denaturation at 98 °C for 20 s, annealing at 50–60 °C for 15 s (−1 °C per cycle), followed by elongation at 72 °C for 15 s, and 2 cycles of denaturation at 98 °C for 20 s, annealing at 50 °C for 15 s, followed by elongation at 72 °C for 15 s. The final extension was carried out at 72 °C for 5 min. In the following second PCR, the ITS2 region was amplified with primer pair fITS7^58^ and ITS4 containing the Illumina adapter sequences using the 1:10 diluted products of the previous reaction. PCR thermo-cycle conditions were as follows: initial denaturation at 95 °C for 5 min, 25 cycles of denaturation at 98 °C for 20 s, annealing at 56 °C for 15 s, followed by elongation at 72 °C for 15 s, and a final extension at 72 °C for 5 min. We purified the PCR products with an Agencourt AMPure XP kit (Beckman Coulter, Krefeld, Germany). Adapters and barcodes were introduced to the samples using Illumina Nextera XT Indices (Illumina Inc., San Diego, CA, USA). The final PCR products were purified again with AMPure beads and then quantified by PicoGreen assays (Molecular Probes, Eugene, OR, USA). Samples were pooled in equimolar concentration and used for paired-end sequencing (2 × 300 bp) on an Illumina MiSeq (Illumina Inc., San Diego, CA, USA) at the Department of Soil Ecology, Helmholtz Centre for Environmental Research – UFZ (Halle, Germany).

### Bioinformatics workflow

Raw forward and reverse reads were de-multiplexed by the Illumina MiSeq Reporter software package v2.5.1.3 with default parameters (mismatch = 4) and then processed using custom bash scripts on a high performance computing (HPC) cluster. We used the workflow presented in Schöps, *et al*.^12^. Briefly, paired-end reads were merged using PandaSeq v2.8.1^59^. To retain high-quality reads, reads with an average quality score (Phred score) of <24, shorter than 200 nt, with any ambiguous nucleotide, and with homopolymers of 10 nt or longer were removed using MOTHUR v1.39.5^60^. The reads were then pre-clustered using cd-hit-454 v4.6.1^61^ and putative chimeric sequences were discarded after a chimera check using UCHIME^62^ as implemented in MOTHUR^60^. Afterwards, reads from each sample were pooled together, de-replicated into unique sequences, and sorted by decreasing abundance using OBITools v1.2.11^63^. OTU clustering was performed based on 3% sequence dissimilarity using vsearch v2.2.0^64^ followed by a chimera check on the representative OTU sequences (the most abundant sequence in each OTU) using UCHIME. The OTU representative sequences were taxonomically assigned against the reference sequences from the UNITE database v7^65^ using the naïve Bayesian classifier (default) as implemented in MOTHUR^60^. The taxonomic classification of Basidiobolomycota, Kickxellomycota, Mucoromycota, Glomeromycota, and Mortierellomycota was based on Tedersoo, *et al*.^66^. OTUs that comprised only singletons, doubletons and tripletons were not subjected to further analyses (Supplementary Table S5). Potential fungal trophic modes were assigned to the fungal OTUs when possible by using the functional annotation tool: FUNGuild^21^.

### Data analysis

All statistical analyses and graphs were made in R v3.5.1^67^. We generated rarefactions curves using ‘rarefy’ function from the vegan package^68^ to assess the coverage of the sequencing depth (Supplementary Fig. S2). The Venn and Euler diagrams were created using R packages limma^69^ and eulerr^70^, respectively. For the α-diversity analysis the OTU table was randomly normalized to the smallest number of reads per sample (27,468 reads). Estimates of α-diversity (observed and estimated (abundance-based coverage estimator – ACE) OTU richness, Shannon index, and evenness) were calculated using vegan and fossil^71^. We tested all α-diversity indices for normality and homogeneity of variance using the Shapiro-Wilk test in base R and the Levene’s test in the car package^72^, respectively. Differences in α-diversity of rhizosphere fungi between plant species, plant type, and LUI (*P < *0.05) were tested by performing linear mixed effect models (LMEM, packages lmer^73^ and lmerTest^74^) including experimental plot nested in soil type as random factor. We calculated marginal and conditional *R*^2^-values to evaluate goodness-of-fit of the model using the ‘r.squaredGLMM’ function^75^. For analysis of the community composition (β-diversity), we normalized the OTU table using the “trimmed means of M” (TMM) method with the package edgeR^76,77^. The effect of plant species, plant type and LUI on fungal community composition was assessed by performing permutational multivariate analysis of variance (PERMANOVA) with 10^3^ permutations using the function ‘adonis’ from the vegan package^68^. Relationships of fungal communities of plant types and plant species were visualized using two-dimensional non-metric multidimensional scaling (NMDS) based on Bray-Curtis dissimilarity index in the vegan package. Topographic features (elevation and slope), geographic location (latitude and longitude of the experimental plots), and climate variables (relative humidity, soil moisture, air temperature at 10 and 200 cm) were fitted post-hoc to the NMDS plots using the ‘envfit’ function in vegan^68^. The multivariate homogeneity of group dispersions was tested using ‘betadisper’ function followed by ‘permutest’ (both vegan package) to determine whether variances differed between phytometer and resident plants. Similarity percentages (SIMPER) analysis was used to discriminate fungal genera between the plant types (phytometer vs. resident plants) using the ‘simper’ function and Bray-Curtis dissimilarity in the vegan package. We extracted the top 30 fungal genera, which add up together with unclassified fungi to a total of 76% of the observed dissimilarities between the plant types, for further analysis of their ‘land-use niche’^19^. To characterize the ecological niches, we determined the ‘land-use niche’ for the top 30 fungal genera and analyzed the data with a randomization approach according to Chisté, *et al*.^19^. The ‘niche optimum’ of a fungal genus *j* was calculated by defining the abundance-weighted means (*μ*_*j*_) of the land-use gradient values:

2 $${\mu }_{j}=\mathop{\sum }\limits_{s=1}^{13}{p}_{j,s}\times {L}_{s^{\prime} }$$

where *p* is the proportion of fungal genus j on site s (compared to its total abundance on each site) and *L* is the LUI on site *s*. The abundance-weighted standard deviation (*σ*_*j*_) of LUI of each genus reflected the ‘niche breadth’. The *μ*_*j*_ were compared to a null model assuming that each fungal genus can occur on every site along the LUI gradient with the same probability. The null model calculated after 10,000 iterations an expected *μ*_*j*_, which chooses LUI of random sites for each fungal genus and considers the number of sites each fungal genus occurred^38^. *P* values were calculated by relating the 10,000 expected *μ*_*j*_ from the null models to the observed *μ*_*j*_. Fungal genera were termed “winners,” when *μ*_*j,obs*_ > *μ*_*j,exp*_ (*P* < 0.05), whereas genera were termed “losers,” when *μ*_*j,obs*_ < *μ*_*j,exp*_ (*P* < 0.05). For the remaining genera, which could not be assigned to the two categories, it was tested whether they are specialized on intermediate land-use intensities (“mid-specialists”; quantification of their niche breadth). Therefore, their observed and expected weighted coefficient of variation (CV_*j*_ = *σ*_*j*_/*μ*_*j*_) was calculated, whereby specialists of intermediate land-use intensity would have CV_*j,obs*_ < CV_*j,exp*_ and an observed *μ*_*j*_ similar to expected *μ*_*j*_. Fungal genera that were not assigned to one of the three categories and with no considerable deviance from the null models were named “neutrals”. We assigned the trophic mode of the top 30 fungal genera by parsing them against the open FUNGuild database^21^. The trophic mode of the genus *Paraphoma* was altered to “pathotroph”^78,79^. For the sake of completeness fungal genera designated in more than one trophic mode are admittedly shown in the results, but their functions were not interpreted further.

## Supplementary information

Electronical supplementary material.

Supplementary Table S5.

## Data Availability

All sequence data generated in this study have been deposited in the Sequence Read Archive (SRA; SRR9281775-SRR9281826) with the accession number PRJNA433935. Data generated or analyzed during this study are included in this published article and its supplementary information files.
