# Supplementary material for: Resident and phytometer plants host comparable rhizosphere fungal communities in managed grassland ecosystems
Source: Sci Rep. 2020 Jan 22;10:919. doi: 10.1038/s41598-020-57760-x (PMC6976665; doi:10.1038/s41598-020-57760-x)
Supplement: Supplementary file 1 — Electronical supplementary material. [file 41598_2020_57760_MOESM1_ESM.pdf]

Supplementary information

Authors: Ricardo Schöps[1,2,*], Kezia Goldmann[1], Lotte Korell[3,4,5], Helge Bruelheide[3,5], Tesfaye Wubet[4,5] & François Buscot[1,5]

Title: Resident and phytometer plants host comparable rhizosphere fungal communities in managed grassland ecosystems

Author affiliation:

[1]UFZ – Helmholtz Centre for Environmental Research, Department of Soil Ecology, Theodor-Lieser-Straße 4, 06120 Halle/Saale, Germany

[2]University of Leipzig, Department of Biology II, Johannisallee 21, 04103 Leipzig, Germany

[3]Martin Luther University Halle-Wittenberg, Institute of Biology/Geobotany and Botanical Garden, Am Kirchtor 1, 06108 Halle/Saale, Germany

[4]UFZ – Helmholtz-Centre for Environmental Research, Department of Community Ecology, Theodor-Lieser-Straße 4, 06120 Halle/Saale, Germany

[5]German Centre for Integrative Biodiversity Research (iDiv) Halle-Jena-Leipzig, Deutscher Platz 5e, 04103 Leipzig, Germany

***Correspondence:** Ricardo Schöps; schoeps.ric@gmail.com

Supplementary Table S1. Diversity indices for fungal communities. Strong differences are indicated in bold (*P* < 0.05). Soil type nested in experimental plot was the random factor

	Richness			ACE			Shannon			Evenness		
	df	*F*	*P*	df	*F*	*P*	df	*F*	*P*	df	*F*	*P*
LUI	1, 13	1.475	0.246	1, 13	1.375	0.262	1, 13	1.127	0.308	1, 13	0.105	0.751
Plant species	1, 39	8.389	**0.006**	1, 39	10.111	**0.003**	1, 39	3.538	0.067	1, 39	1.043	0.314
Plant type	1, 39	1.846	0.182	1, 39	2.950	0.094	1, 39	0.915	0.345	1, 39	0.282	0.598
Species:Type	1, 39	0.495	0.486	1, 39	0.687	0.412	1, 39	3.356	0.075	1, 39	5.702	**0.022**
Observations	52			52			52			52		
Marg. R^2	0.17			0.20			0.15			0.11		
Cond. R^2	0.38			0.41			0.19			0.26		

LUI, land-use intensity; Plant species, *Dactylis glomerata* versus *Plantago lanceolata*; Plant type, resident plants versus phytometer plants; ACE, Abundance-based coverage estimator; Species:Type, Plant species:plant type; df, degrees of freedom numerator and degrees of freedom denominator; *F* and *P*, *F*-value and *P*-value. Marginal R^2 (marg.) indicates how much variance is explained in the model without random factors, conditional R^2 (cond.) is how much variance is explained by the model including random factors

Supplementary Table S2. The first 50 fungal genera that contribute the most to the observed pairwise variation in the composition of the rhizosphere mycobiomes of the different plant types based on analysis of percentage similarity (SIMPER). The top 30 fungal genera are highlighted in grey which add up together with unclassified fungi to a total of 76 % of the observed dissimilarities

Overall average dissimilarity = 53 %		Abundance	Abundance
Fungal genus	Contribution	Phytometer	Resident
unclassified	12.34	0.30	0.30
Mortierella	9.15	0.10	0.09
Mycosphaerella	6.30	0.06	0.08
Cryptococcus	5.63	0.07	0.08
Camarophyllopsis	3.45	0.01	0.03
Gibberella	3.12	0.03	0.02
Monographella	3.07	0.01	0.03
Ramariopsis	2.32	0.01	0.02
Conocybe	2.32	0.02	0.01
Trichosporon	2.31	0.02	0.01
Preussia	2.02	0.02	0.01
Holtermannia	1.89	0.02	0.02
Clavulinopsis	1.86	0.01	0.01
Didymella	1.81	0.02	0.02
Serendipita	1.71	0.02	0.01
Tricholoma	1.69	0.02	0.00
Coprinopsis	1.42	0.01	0.01
Auricularia	1.35	0.01	0.01
Paraphoma	1.31	0.01	0.01
Mycena	1.26	0.00	0.01
Hygrocybe	1.04	0.00	0.01
Cystofilobasidium	1.04	0.01	0.01
Tetracladium	1.03	0.01	0.01
Clavaria	1.00	0.00	0.01
Calyptella	0.99	0.01	0.00
Heteroconium	0.91	0.01	0.01
Entoloma	0.86	0.01	0.00
Pyrenochaetopsis	0.86	0.01	0.01
Ceratobasidium	0.85	0.00	0.01
Articulospora	0.76	0.01	0.01
Deconica	0.69	0.01	0.00
Stropharia	0.69	0.00	0.01
Rhizophlyctis	0.62	0.00	0.00
Hannaella	0.60	0.01	0.00
Dioszegia	0.60	0.01	0.01
Metarhizium	0.55	0.01	0.00
Podospora	0.55	0.00	0.00
Exophiala	0.55	0.01	0.01
Cuphophyllus	0.52	0.00	0.01
Septoria	0.50	0.00	0.00
Schwanniomyces	0.47	0.00	0.00
Thanatephorus	0.48	0.00	0.00
Coprinus	0.45	0.00	0.00
Pyrenochaeta	0.44	0.00	0.00
Chaetosphaeronema	0.41	0.00	0.00
Lecythophora	0.41	0.00	0.00
Mycocentrospora	0.39	0.00	0.00
Myrothecium	0.37	0.00	0.00
Colletotrichum	0.35	0.00	0.00
Meliniomyces	0.35	0.00	0.00

Supplementary Table S3. The 'winner', 'mid-specialists', and 'losers' of intensive land-use of the top 30 fungal genera

Trophic mode	Genera	Total	Phytometer		Resident	
			Da.gl	Pl.la	Da.gl	Pl.la
Sym	[B]*Serendipita*	neutral	neutral	neutral	neutral	neutral
Sap	[B]*Calyptella*	winner	neutral	neutral	neutral	neutral
Sap	[B]*Auricularia*	neutral	neutral	neutral	neutral	neutral
Sap	[B]*Coprinopsis*	neutral	neutral	neutral	neutral	neutral
Sap	[A]*Preussia*	neutral	neutral	neutral	neutral	neutral
Sap	[A]*Articulospora*	neutral	neutral	neutral	neutral	neutral
Sap	[A]*Heteroconium*	neutral	neutral	neutral	neutral	neutral
Sap	[B]*Conocybe*	neutral	neutral	winner	neutral	winner
Sap	[B]*Cystofilobasidium*	neutral	neutral	neutral	neutral	mid-spec
Sap	[A]*Tetracladium*	mid-spec	neutral	neutral	neutral	neutral
Sap	[B]*Clavulinopsis*	neutral	neutral	loser	neutral	mid-spec
Sap	[B]*Clavaria*	loser	mid-spec	neutral	loser	loser
Sap	[B]*Deconica*	neutral	neutral	neutral	neutral	neutral
Sap	[B]*Ramariopsis*	loser	loser	loser	loser	loser
Sap	[B]*Camarophyllopsis*	loser	loser	neutral	loser	loser
Pat	[A]*Paraphoma*	winner	winner	neutral	neutral	winner
Pat	[A]*Monographella*	winner	winner	winner	neutral	neutral
Pat	[A]*Gibberella*	mid-spec	neutral	neutral	neutral	neutral
Pat	[A]*Mycosphaerella*	neutral	neutral	neutral	neutral	neutral
Pat	[B]*Trichosporon*	neutral	neutral	loser	neutral	neutral
Sap/Sym	[M]*Mortierella*	neutral	neutral	neutral	neutral	neutral
Sap/Sym	[B]*Hygrocybe*	loser	neutral	neutral	neutral	neutral
Pat/Sym	[B]*Tricholoma*	neutral	neutral	neutral	neutral	neutral
Pat/Sap	[A]*Didymella*	mid-spec	neutral	mid-spec	mid-spec	neutral
Pat/Sap	[B]*Mycena*	loser	neutral	loser	mid-spec	mid-spec
Pat/Sap/Sym	[B]*Entoloma*	neutral	neutral	neutral	winner	neutral
Pat/Sap/Sym	[B]*Ceratobasidium*	neutral	neutral	neutral	neutral	neutral
Pat/Sap/Sym	[B]*Cryptococcus*	neutral	mid-spec	neutral	neutral	neutral
Pat/Sap/Sym	[A]*Pyrenochaetopsis*	neutral	neutral	neutral	neutral	neutral
unknown	[B]*Holtermannia*	neutral	mid-spec	neutral	neutral	neutral

The abundance-weighted means ($\mu_{j,obs}$) and standard deviations ($\sigma_{j,obs}$) of land-use intensity (LUI; consisting of the land-use elements grazing, mowing, and fertilization) were calculated for each of the top 30 fungal genera. A fungal genus was termed 'loser' of intensive land-use when the $\mu_{j,obs}$ was significantly smaller than the expected ($\mu_{j,exp}$; $P < 0.05$) by the null model with a randomization approach (10,000 iterations). In turn, a 'winner' had a significantly higher $\mu_{j,obs}$ than $\mu_{j,exp}$ ($P < 0.05$). In addition, fungal genera with a smaller observed weighted coefficient of variation than expected ($CV_{j,obs} = \sigma_{j,obs}/\mu_{j,obs} < CV_{j,exp} = \sigma_{j,exp}/\mu_{j,exp}$) and $\mu_{j,obs} = \mu_{j,exp}$ ($P > 0.05$) were named 'mid-specialists' (mid-spec). Intensive land-use showed no influence on fungal genera that were termed 'neutral'. Da.gl, *Dactylis glomerata*; Pl.la, *Plantago lanceolata*; Fungal trophic modes: Sym – symbiotroph, Sap – saprotroph, Pat – pathotroph, Sap/Sym – saprotroph/symbiotroph, Pat/Sap – pathotroph/saprotroph, Pat/Sap/Sym - pathotroph / saprotroph/symbiotroph; [A], ascomycetous genus; [B], basidiomycetous genus; [M], Mortierellomycota

Supplementary Table S4. Information to environmental variables of the 13 experimental plots (EPs).

EP	LUI index	Land-use elements			Soil type	Elevation	Slope	rH	SM	Ta 10	Ta 200
		G	M	F							
AEG01	1.79	0.00	1.73	1.46	Leptosol	691.03	2.85	85.344	29.464	10.080	9.049
AEG05	1.77	0.98	0.82	1.33	Leptosol	717.79	2.79	89.677	46.892	10.502	9.192
AEG08	1.32	1.02	0.73	0.00	Leptosol	760.28	3.74	81.701	42.280	10.769	9.307
AEG15	2.44	0.00	2.45	3.51	Leptosol	746.77	6.81	82.233	46.107	11.291	9.789
AEG16	1.62	1.01	1.09	0.52	Leptosol	723.85	5.82	87.195	37.725	10.977	9.403
AEG18	2.18	0.00	2.09	2.65	Leptosol	765.07	3.76	80.877	42.416	10.588	9.360
AEG21	3.62	8.18	0.27	4.64	Leptosol	732.75	3.02	82.851	38.738	10.261	9.671
AEG22	1.25	0.35	0.82	0.38	Leptosol	796.64	8.47	85.571	46.639	10.455	9.426
AEG31	1.36	1.29	0.55	0.00	Leptosol	728.03	8.12	90.922	37.066	10.473	9.827
AEG33	1.32	1.75	0.00	0.00	Leptosol	801.64	5.20	82.340	45.013	10.321	9.309
AEG38	1.35	0.00	1.64	0.19	Cambisol	815.76	1.73	80.484	46.824	11.099	9.912
AEG44	1.86	3.45	0.00	0.00	Cambisol	682.37	13.88	89.603	49.107	10.395	9.209
AEG45	1.38	0.00	1.82	0.09	Cambisol	733.72	4.32	86.149	44.665	10.167	9.037

LUI, land-use intensity for the interval 2006-2014 and for the calculation see Methods: Site description; land-use elements: F – fertilization level (kg N $ha^{-1}yr^{-1}$), M – mowing frequency (cuts yr^{-1}), G – grazing intensity (livestock units days of grazing $ha^{-1}yr^{-1}$); soil type, according to the Food and Agriculture Organization (FAO) soil classification system; rH, percentage of relative humidity (0-100 %) in 200 cm aboveground; SM, percentage of soil moisture (volumetric water content) in 10 cm depth; Ta 10, air temperature in 10 cm aboveground (degree Celcius, -40°C to 60°C); Ta 200, air temperature in 200 cm aboveground (degree Celcius, -40°C to 60°C.

Supplementary Table S5. The CSV table contains the unrarefied operational taxonomic unit table with taxonomic assignment used for this study.

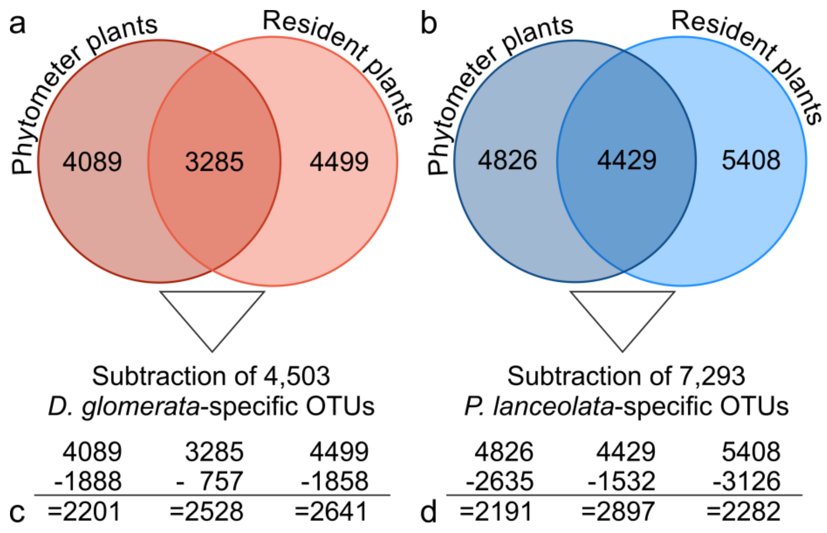

Supplementary Figure S1. Numbers of fungal OTUs in the rhizosphere of **a** *Dactylis glomerata* and **b** *Plantago lanceolata* shared between the plant types (phytometer vs. resident plants). The plant species-specific OTUs (see Fig. 1b) of **c** *D. glomerata* (4,503 OTUs) and **d** *P. lanceolata* (7,293 OTUs) were subtracted from the phytometer plant, resident plant, and shared sets. The remaining OTUs occurred in the rhizosphere of both plant species.

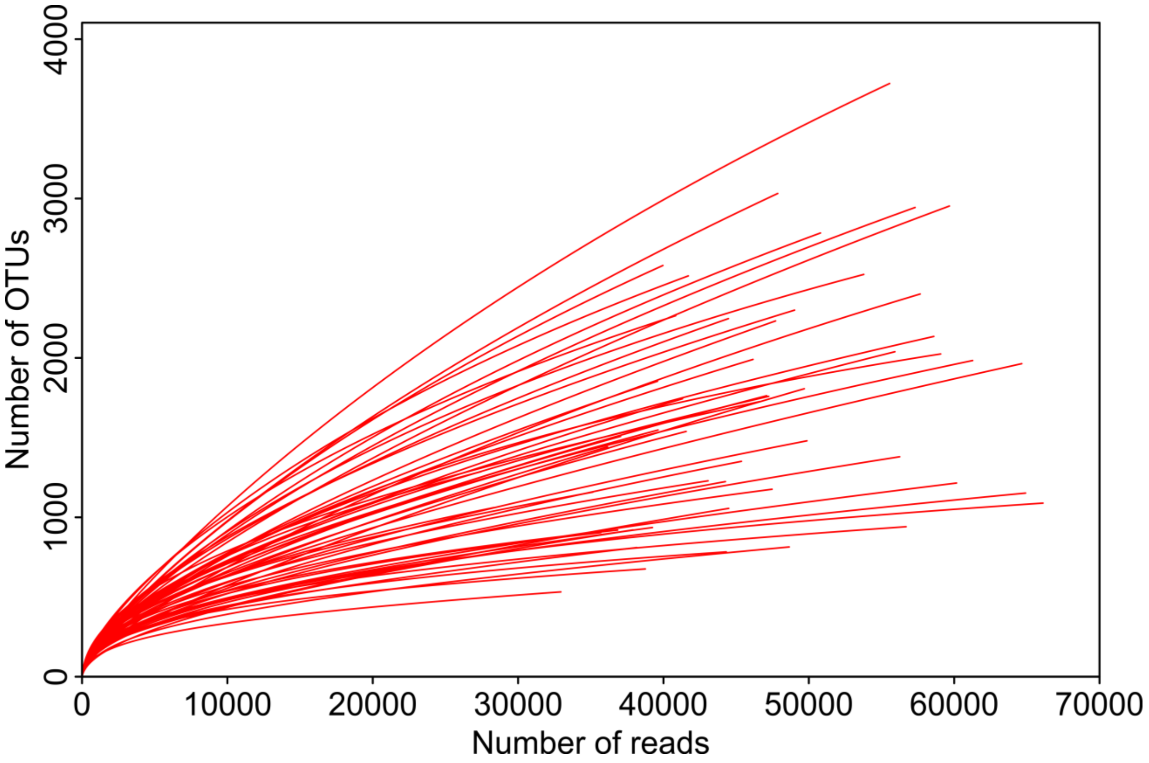

Supplementary Figure S2. Rarefaction curves illustrating the relationship between number of reads per sample and fungal species richness in operational taxonomic units (OTUs)
